# Incremental parameter estimation of kinetic metabolic network models

**DOI:** 10.1186/1752-0509-6-142

**Published:** 2012-11-21

**Authors:** Gengjie Jia, Gregory Stephanopoulos, Rudiyanto Gunawan

**Affiliations:** 1 Chemical and Pharmaceutical Engineering, Singapore-MIT Alliance, Singapore, 117576, Singapore; 2 Department of Chemical Engineering, Massachusetts Institute of Technology, Cambridge, MA, 02139, USA; 3 Institute for Chemical and Bioengineering, ETH Zürich, 8093, Zürich, Switzerland

**Keywords:** Incremental parameter estimation, Kinetic modeling, Metabolic network, GMA model

## Abstract

**Background:**

An efficient and reliable parameter estimation method is essential for the creation of biological models using ordinary differential equation (ODE). Most of the existing estimation methods involve finding the global minimum of data fitting residuals over the entire parameter space simultaneously. Unfortunately, the associated computational requirement often becomes prohibitively high due to the large number of parameters and the lack of complete parameter identifiability (i.e. not all parameters can be uniquely identified).

**Results:**

In this work, an incremental approach was applied to the parameter estimation of ODE models from concentration time profiles. Particularly, the method was developed to address a commonly encountered circumstance in the modeling of metabolic networks, where the number of metabolic fluxes (reaction rates) exceeds that of metabolites (chemical species). Here, the minimization of model residuals was performed over a subset of the parameter space that is associated with the degrees of freedom in the dynamic flux estimation from the concentration time-slopes. The efficacy of this method was demonstrated using two generalized mass action (GMA) models, where the method significantly outperformed single-step estimations. In addition, an extension of the estimation method to handle missing data is also presented.

**Conclusions:**

The proposed incremental estimation method is able to tackle the issue on the lack of complete parameter identifiability and to significantly reduce the computational efforts in estimating model parameters, which will facilitate kinetic modeling of genome-scale cellular metabolism in the future.

## Background

The estimation of unknown kinetic parameters from time-series measurements of biological molecules is a major bottleneck in the ODE model building process in systems biology and metabolic engineering [1]. The majority of current estimation methods involve simultaneous (single-step) parameter identification, where model prediction errors are minimized over the entire parameter space. These methods often rely on global optimization methods, such as simulated annealing, genetic algorithms and other evolutionary approaches [1-3]. The problem of obtaining the best-fit parameter estimates however, is typically ill-posed due to issues related with data informativeness, problem formulation and parameter correlation, all of which contribute to the lack of complete parameter identifiability. Not to mention, finding the global minimum of model residuals over highly multidimensional parameter space is challenging and can become prohibitively expensive to perform on a computer workstation, even for tens of parameters.

Here, we consider the modeling of cellular metabolism using the canonical power-law formalism, specifically the generalized mass action (GMA) systems [4,5]. The power-law formalism has many advantages, which have been detailed elsewhere [1,6]. Notably, power laws have a relatively simple structure that permits algebraic manipulation in the logarithmic scale, but nonetheless is capable of describing essentially any nonlinearity. Regulatory interactions among metabolites can also be described straightforwardly through the kinetic order parameters, establishing an equivalence between structural identification and parametric estimation. However, the number of parameters increases proportionally with the number of metabolites and fluxes, leading to a large-scale parameter identification problem, one where single-step estimation methods often struggle to converge.

The integration of ODE often constitutes a major part of the computational cost in the parameter estimation, especially when the ODE model is stiff [7]. While stiffness can genuinely arise due to a large time scale separation of the reaction kinetics in the real system, stiff ODEs could also result from unrealistic combinations of parameter values during the parameter optimization procedure, especially when a global optimizer is used. The parameter estimation of ODE models using power-law kinetics is particularly prone to stiffness problem since many of the unknown parameters are the exponents of the concentrations. For this reason, alternative formulations have been proposed that avoid these ODE integrations either completely [7,8] or partially [9-11]. Particularly, computational cost could be significantly reduced by decomposing the estimation problem into two phases, starting with the calculation of dynamic reaction rates or fluxes from the slopes of concentration data, followed by the least square regressions of kinetic parameters [12-14]. In this case, the final parameter estimation is done one flux at a time, each involving only a handful of parameters and thus, the global minimum solution can be either computed analytically (for example, when using log-linear power-law flux functions) or determined efficiently. Moreover, as the first estimation phase (flux estimation) depends only on the assumption of the topology of the metabolic network, the flux estimates can subsequently be used to guide the selection of the most appropriate flux functions for the second phase or to detect inconsistencies in the assumed topology of the network separately from the flux equations [14]. However, the application of this method requires the number of metabolites to be equal to or larger than that of fluxes, so that the flux estimation can result in a unique solution. Since the reverse situation is more commonly encountered in the typical metabolic networks, a generalization of this incremental estimation approach becomes the main focus in this study.

As noted above, the new parameter estimation method in this work is built on the concept of incremental identification [12,13] or dynamical flux estimation (DFE) method [14,15]. The proposed method provides two new contributions: (1) an ability to handle the more general scenario, where the number of reactions exceeds that of the metabolites and (2) high numerical efficiency through the reduction of the parameter search space. Specifically, two parameter estimation formulations are proposed with objective functions that depend on model prediction errors of metabolite concentrations and of concentration time-slopes. An extension of this strategy to circumstances where concentration data of some metabolites are missing is also presented. The proposed method is applied to two previously published GMA models and compared with single-step estimation methods, in order to demonstrate its efficacy.

## Methods

The generalized mass action model of cellular metabolism describes the mass balance of metabolites, taking into account all metabolic influxes and effluxes and their stoichiometric ratios, as follows:

(1) $$dX\left(t,p\right)/\mathrm{dt}=\dot{X}\left(t,p\right)=Sv\left(X,p\right)\text{,}$$

where **X**(*t*,**p**) is the vector of metabolic concentration time profiles, **S** ∈ **R**^*m* × *n*^ is the stoichiometric matrix for *m* metabolites that participate in *n* reactions, and **v**(**X**,**p**) denotes the vector of metabolic fluxes (i.e. reaction rates). Here, each flux is described by a power-law equation:

(2) $$v_{j}\left(X,p\right)=\gamma_{j}\prod_{i}X_{i}^{f_{\mathrm{ji}}}\text{,}$$

where *γ*_*j*_ is the rate constant of the *j*-th flux and *f*_*ji*_ is the kinetic order parameter, representing the influence of metabolite *X*_*i*_ on the *j*-th flux (positive: *X*_*i*_ is an activating factor or a substrate, negative: *X*_*i*_ is an inhibiting factor). In incremental parameter identification, a data pre-processing step (e.g. smoothing or filtering) is usually applied to the noisy time-course concentration data **X**_*m*_(*t*_*k*_), in order to improve the time-slope estimates ${\dot{X}}_{m}\left(t_{k}\right)$. Subsequently, the dynamic metabolic fluxes **v**(*t*_*k*_) are estimated from Equation (1) by substituting $\dot{X}\left(t\right)$ with ${\dot{X}}_{m}\left(t_{k}\right)\text{.}$ Finally, the kinetic parameters associated with the *j*-th flux (i.e. *γ*_*j*_ and *f*_*ji*_’s) can be calculated using a least square regression of the power law flux function in Equation (2) against the estimated *v*_*j*_(*t*_*k*_). Note that for GMA models, the least square parameter regressions in the last step are linear in the logarithmic scale and thus, can be performed very efficiently.

A unique set of dynamic flux values **v**(*t*_*k*_) can only be computed from ${\dot{X}}_{m}\left(t_{k}\right)=Sv\left(t_{k}\right)\text{,}$ when the number of metabolites exceeds that of fluxes. However, a metabolite in general can participate in more than one metabolic flux (*m* < *n*). In such a situation, there exist an infinite number of dynamic flux combinations **v**(*t*_*k*_) that satisfy ${\dot{X}}_{m}\left(t_{k}\right)=Sv\left(t_{k}\right)\text{.}$ The dimensionality of the set of flux solutions is equal to the degree of freedom (DOF), given by the difference between the number of fluxes and the number of metabolites: *n*_*DOF*_ = *n-m* >0 (assuming **S** has a full row rank, i.e. there is no redundant ODE in Equation (1)). The positive DOF means that the values of *n*_*DOF*_ selected fluxes can be independently set, from which the remaining fluxes can be computed. This relationship forms the basis of the proposed estimation method, in which the model goodness of fit to data is optimized by adjusting only a subset of parameters associated with the independent fluxes above.

Specifically, we start by decomposing the fluxes into two groups: **v**(*t*_*k*_) = [ **v**_*I*_(*t*_*k*_)^*T*^**v**_*D*_(*t*_*k*_)^*T*^ ]^*T*^ , where the subscripts *I* and *D* denote the independent and dependent subset, respectively. Then, the parameter vector **p** and the stoichiometric matrix **S** can be structured correspondingly as **p** = [ **p**_*I*_**p**_*D*_ ] and **S** = [ **S**_*I*_**S**_*D*_ ]. The relationship between the independent and dependent fluxes can be formulated by rearranging ${\dot{X}}_{m}\left(t_{k}\right)=Sv\left(t_{k}\right)$ into:

(3) $$v_{D}\left(t_{k}\right)={S_{D}}^{-1}\left[{\dot{X}}_{m}\left(t_{k}\right)-S_{I}v_{I}\left(X_{m}\left(t_{k}\right),p_{I}\right)\right]\text{.}$$

In this case, given **p**_*I*_, one can compute the independent fluxes **v**_*I*_(**X**_*m*_(*t*_*k*_),**p**_*I*_) using the concentration data **X**_*m*_(*t*_*k*_), and subsequently obtain **v**_*D*_(*t*_*k*_) from Equation (3). Finally, **p**_*D*_ can be estimated by a simple least square fitting of **v**_*D*_(**X**_*m*_(*t*_*k*_),**p**_*D*_) to the computed **v**_*D*_(*t*_*k*_) one flux at a time, when there are more time points than the number of parameters in each flux.

**Figure 1:**
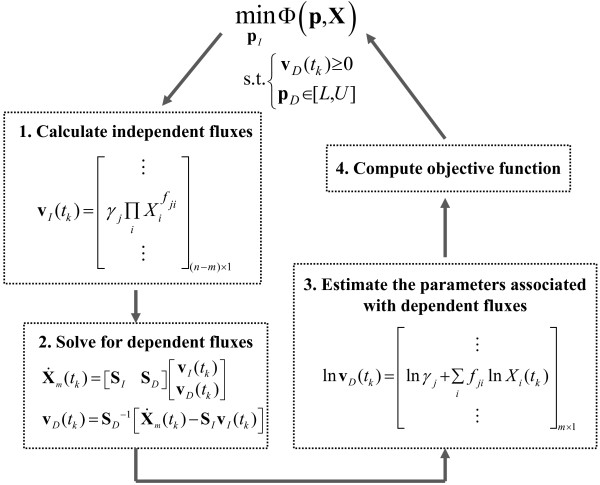
Flowchart of the incremental parameter estimation.

In this study, two formulations of the parameter estimation of ODE models in Equation (1) are investigated, involving the minimization of concentration and slope errors. The objective function for the concentration error is given by

(4) $$\Phi_{C}\left(p,X\right)=\sqrt{\frac{1}{mK}\sum_{k=1}^{K}{\left[X_{m}\left(t_{k}\right)-X\left(t_{k},p\right)\right]}^{T}\left[X_{m}\left(t_{k}\right)-X\left(t_{k},p\right)\right]}$$

and that for the slope error is given by

(5) $$\Phi_{S}\left(p,X\right)=\sqrt{\begin{array}{c} \frac{1}{mK}\sum_{k=1}^{K}{\left[{\dot{X}}_{m}\left(t_{k}\right)-Sv\left(X_{m}\left(t_{k}\right),p\right)\right]}^{T}\\ \left[{\dot{X}}_{m}\left(t_{k}\right)-Sv\left(X_{m}\left(t_{k}\right),p\right)\right]\text{,} \end{array}}$$

where *K* denotes the total number of measurement time points and **X**(*t*_*k*_,**p**) is the concentration prediction (i.e. the solution to the ODE model in Equation (1)). Figure 1 describes the formulation of the incremental parameter estimation and the procedure for computing the objective functions. Note that the computation of Φ_C_ requires an integration of the ODE model and thus, the estimation using this objective function is expected to be computationally costlier than that using Φ_S_. On the other hand, metabolic mass balance is only approximately satisfied at discrete time points *t*_*k*_ during the parameter estimation using Φ_S_, as the ODE model is not integrated.

There are several important practical considerations in the implementation of the proposed method. The first consideration is on the selection of the independent fluxes. Here, the set of these fluxes is selected such that (i) the *m* × *m* submatrix **S**_*D*_ is invertible, (ii) the total number of the independent parameters **p**_*I*_ is small, and (iii) the prior knowledge of the corresponding **p**_*I*_ is maximized. The last two aspects should lead to a reduction in the parameter search space and the cost of finding the global optimal solution of the minimization problem in Figure 1. The second consideration is regarding constraints in the parameter estimation. Biologically relevant values of parameters are often available, providing lower and/or upper bounds for the parameter estimates. In addition, enzymatic reactions in the ODE model are often assumed to be irreversible and thus, dynamic flux estimates are constrained to be positive. Hence, the parameter estimation involves a constrained minimization problem, for which many global optimization algorithms exist.

So far, we have assumed that the time-course concentration data are available for all metabolites. However, the method above can be modified to accommodate more general circumstances, in which data for one or several metabolites are missing. In this case, the ODE model is first rewritten to separate the mass balances associated with measured and unmeasured metabolites, such that

(6) $$\dot{X}\left(t,p\right)=\left[\begin{array}{c} {\dot{X}}_{M}\\ {\dot{X}}_{U} \end{array}\right]\left(t,p\right)=\left[\begin{array}{c} S_{M}\\ S_{U} \end{array}\right]v\left(X_{M},X_{U},p\right)$$

where the subscripts *M* and *U* refer to components that correspond to measured and unmeasured metabolites, respectively. Again, if the fluxes are split into two categories **v**_*I*_ and **v**_*D*_ as above, the following relationship still applies for the measured metabolites:

(7) $$v_{D}\left(t_{k}\right)={S_{D,M}}^{-1}\left[{\dot{X}}_{M}\left(t_{k}\right)-S_{I,M}v_{I}\left(t_{k}\right)\right]$$

**Figure 2:**
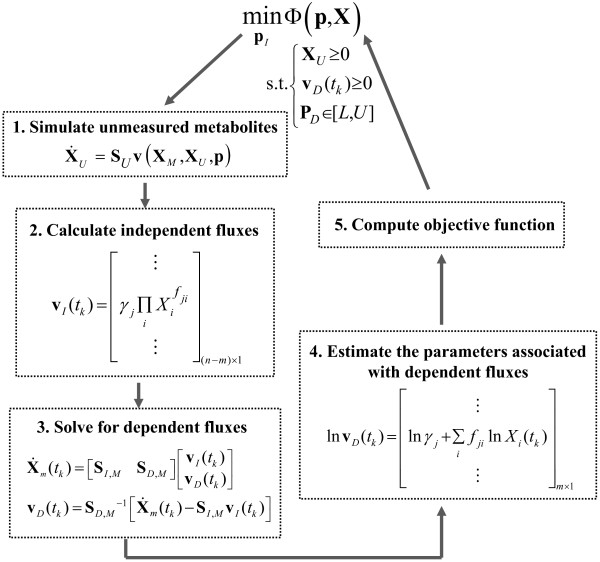
Flowchart of the incremental parameter estimation when metabolites are not completely measured.

Naturally, the degree of freedom associated with the dynamic flux estimation is higher by the number of component in **X**_*U*_ than before. Figure 2 presents a modification of the parameter estimation procedure in Figure 1 to handle the case of missing data, in which an additional step involving the simulation of unmeasured metabolites ${\dot{X}}_{U}=S_{U}v\left(X_{M},X_{U},p\right)$ will be performed. In this integration, **X**_*M*_ is set as an external variable, whose time-profiles are interpolated from the measured concentrations. The set of independent fluxes **v**_*I*_ are now selected to include all fluxes that appear in ${\dot{X}}_{U}$ and those that lead to a full column ranked **S**_*D,M*_. If **S**_*D,M*_ is a non-square matrix, then a pseudo-inverse will be done in Equation (7). Of course, the same considerations mentioned above are equally relevant in this case. Note that the initial conditions of **X**_*U*_ will also need to be estimated.

## Results

Two case studies: a generic branched pathway [7] and the glycolytic pathway of *L. lactis*[16], were used to evaluate the performance of the proposed estimation method. In addition, simultaneous estimation methods employing the same objective functions in Equations (4) and (5) were applied to these case studies, to gauge the reduction in the computational cost from using the proposed strategy. In order to alleviate the ODE stiffness issue, parameter combinations that lead to a violation in the MATLAB (ode15s) integration time step criterion is assigned a large error value (Φ_C_ = 10^3^ for the branched pathway and 10^5^ for the glycolytic pathway). Alternatively, one could also set a maximum allowable integration time and penalize the associated parameter values upon violation, as described above. In this study, the optimization problems were solved in MATLAB using publicly available eSSM GO (Enhanced Scatter Search Method for Global Optimization) toolbox, a population-based metaheuristic global optimization method incorporating probabilistic and deterministic strategies [17,18]. The MATLAB codes of the case studies below are available in Additional file 1. Each parameter estimation was repeated five times to ensure the reliability of the global optimal solution. Unless noted differently, the iterations in the optimization algorithm were terminated when the values of objective functions improve by less than 0.01% or the runtime has exceeded the maximum duration (5 days).

### A generic branched pathway

**Figure 3:**
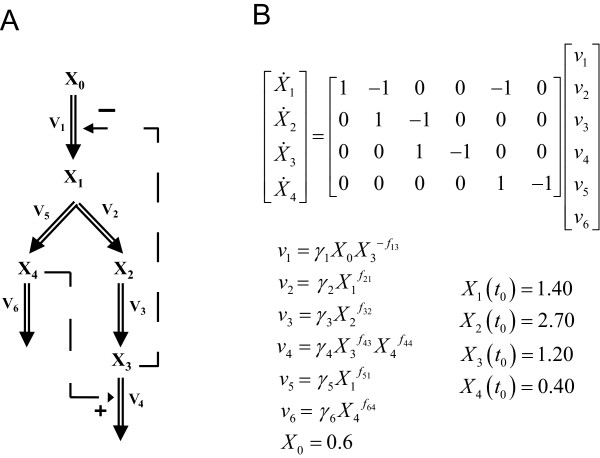
**A generic branched pathway.** (**A**) Metabolic pathway map and (**B**) the GMA model equations [7].

The generic branched pathway in this example consists of four metabolites and six fluxes, describing the transformations among the metabolites (double-line arrows), with feedback activation and inhibition (dashed arrows with plus or minus signs, respectively), as shown in Figure 3A. The GMA model of this pathway is given in Figure 3B, containing a total of thirteen rate constants and kinetic orders. This model with the parameter values and initial conditions reported previously [7] were used to generate noise-free and noisy time-course concentration data (i.i.d additive noise from a Gaussian distribution with 10% coefficient of variation). The noisy data were smoothened using a *6*-th order polynomial, which provided the best relative goodness of fit among polynomials according to Akaike Information Criterion (AIC) [19] and adjusted R^2^[20]. Subsequently, time-slopes of noise-free and smoothened noisy data were computed using the central finite difference approximation.

Here, *v*_*1*_ and *v*_*6*_ were chosen as the independent fluxes as they comprise the least number of kinetic parameters and lead to an invertible **S**_*D*_. The two rate constants and two kinetic orders were constrained to within [0,25] and [0,2], respectively. In addition, all the reactions are assumed to be irreversible.

**Figure 4:**
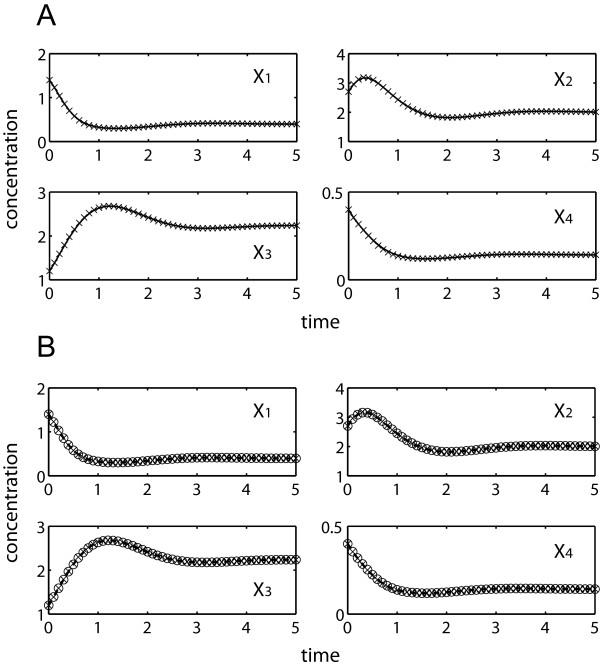
**Simultaneous and incremental estimation of the branched pathway using *in silico *noise-free data (×).** (**A**) concentration predictions using parameter estimates from incremental method by Φ_C_ minimization (–––); (**B**) concentration predictions using parameter estimates from simultaneous method (○) and proposed method (---) by Φ_S_ minimization.

Table 1 compares simultaneous and incremental parameter estimation runs using noise-free data, employing the two objective functions above. Regardless of the objective function, the proposed incremental approach significantly outperformed the simultaneous estimation. When using the concentration-error minimization, simultaneous optimization met great difficulty to converge due to stiff ODE integrations. Only one out of five repeated runs could complete after relaxing the convergence criteria of the objective function to 1%, while the others were prematurely terminated after the prescribed maximum runtime of 5 days. In contrast, the proposed incremental estimation was able to find a minima of Φ_C_ in less than 96 seconds on average with good concentration fit and parameter accuracy (see Figure 4A and Table 1). By avoiding ODE integrations using Φ_S_, the simultaneous estimation of parameters could be completed in roughly 10 minutes duration, but this was much slower than the incremental estimation using Φ_C_. In this case, the incremental method was able to converge in below 2 seconds or over 250 times faster. The goodness of fit to concentration data and the accuracy of parameter estimates were relatively equal for all three completed estimations (see Figure 4B and Table 1). The parameter inaccuracy in this case was mainly due to the polynomial smoothing of the concentration data, since the same estimations using the analytical values of the slopes (by evaluating the right hand side of the ODE model in Equation (1)) could give accurate parameter estimates (see Additional file 2: Table S1).

**Table 1:** Parameter estimations of the branched pathway model using noise-free data

	**Simultaneous method**		**Incremental method**	
	$\text{min}\;\Phi_{C}^{b}$	$\text{min}\;\Phi_{S}^{c}$	**min Φ**_**C**_	**min Φ**_**S**_
CPU time (sec) ^**a**^	56.00 h	620.81 ± 64.30	95.95 ± 11.09	1.56 ± 0.19
eSSM GO iterations	323	4390 ± 391	14 ± 4	10 ± 2
Parameter error (%)	49.10	36.91% ± 1.09	21.56% ± 7.57 × 10^-2^	36.85% ± 6.48 × 10^-3^
$\Phi_{C}^{d}$	4.54 × 10^-3^	6.54 × 10^-3^ ± 5.20 × 10^-5^	4.03 × 10^-3^ ± 6.22 × 10^-8^	6.00 × 10^-3^ ± 5.05 × 10^-7^
$\Phi_{S}^{d}$	7.01 × 10^-2^	2.72 × 10^-2^ ± 1.09 × 10^-5^	3.92 × 10^-2^ ± 9.86 × 10^-6^	2.76 × 10^-2^ ± 4.46 × 10^-10^

a. CPU time was based on a workstation with dual Intel Quad-Core 2.83 GHz processors.

b. Only one out of five runs completed with a relative improvement of the objective function below 1% between iterations. The rest did not converge within the 5-day time limit after iterating for 583, 989, 777, and 661 times. The corresponding Φ_C_ at termination were 4.85× 10^-2^, 1.39 × 10^-2^, 1.75 × 10^-2^ and 3.75 × 10^-2^, respectively.

c. Mean value ± standard deviation out of five repeats.

d. Root mean square error of model predictions, where the underlined value refers to the objective function of the minimization.

**Figure 5:**
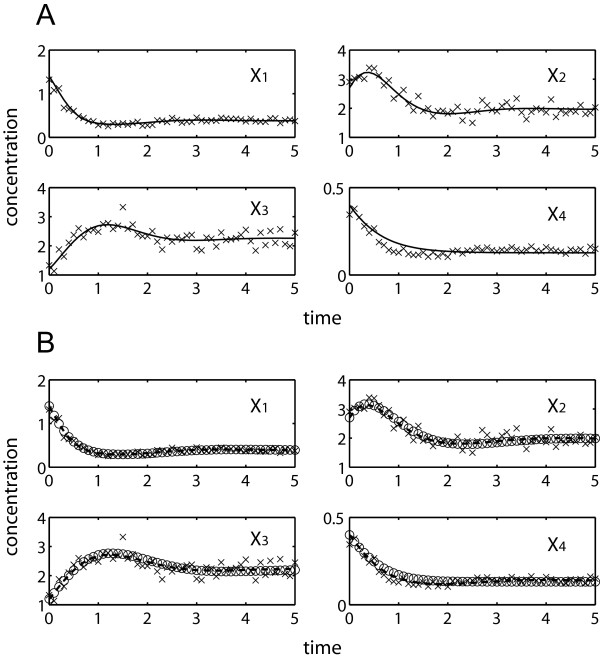
**Simultaneous and incremental estimation of the branched pathway using *in silico *noisy data (×).** (**A**) concentration predictions using parameter estimates from incremental method by Φ_C_ minimization (–––); (**B**) concentration predictions using parameter estimates from simultaneous method (○) and proposed method (---) by Φ_S_ minimization.

Table 2 provides the results of the same estimation procedures as above using noisy data. Data noise led to a loss of information and an expected decline in the parameter accuracy. Like before, the simultaneous estimation using Φ_C_ met stiffness problem and three out of five runs did not finish within the five-day time limit. The incremental approach using either one of the objective functions offered a significant reduction in the computational time over the simultaneous estimation using Φ_S_, while providing comparable parameter accuracy and concentration and slope fit (see Figure 5 and Table 2). In this example, data noise did not affect the computational cost in obtaining the (global) minimum of the objective functions.

**Table 2:** Parameter estimations of the branched pathway model using noisy data

	**Simultaneous method**		**Incremental method**	
	$\text{min}\;\Phi_{C}^{a}$	**min Φ**_***S***_	**min Φ**_***C***_	**min Φ**_***S***_
CPU time (sec)	17.86 h	534.83 ± 22.12	71.88 ± 6.33	1.17 ± 0.12
	44.63 h			
eSSM GO iterations	254	3494 ± 348	12 ± 2	10 ± 3
	426			
Parameter error (%)	75.42	54.36 ± 4.47	75.77 ± 6.11 × 10^-3^	51.15 ± 1.38 × 10^-3^
	34.98			
Φ_*C*_	3.62 × 10^-2^	6.06 × 10^-2^ ± 1.14 × 10^-3^	3.52 × 10^-2^ ± 9.50 × 10^-9^	4.76 × 10^-2^ ± 3.81 × 10^-7^
	3.27 × 10^-2^			
Φ_*S*_	2.06 × 10^-1^	1.34 × 10^-1^ ± 6.02 × 10^-4^	1.64 × 10^-1^ ± 2.23 × 10^-5^	1.38 × 10^-1^ ± 2.26 × 10^-10^
	1.60 × 10^-1^			

a. Two out of five runs completed with a relative improvement of the objective function below 1% between iterations. The rest did not converge within the 5-day time limit after iterating for 805, 699, and 568 times. The corresponding Φ_*C*_ at termination were 4.08 × 10^-2^, 5.05 × 10^-2^ and 6.25 × 10^-2^, respectively.

Finally, the estimation strategy described in Figure 2 was applied to this example using noise-free data and assuming *X*_*3*_ data were missing. Fluxes *v*_*3*_ and *v*_*4*_ that appear in ${\dot{X}}_{3}$ were chosen to be among the independent fluxes and flux *v*_*1*_ was also added to the set such that the dependent fluxes can be uniquely determined from Equation (7). In addition to the parameters associated with the aforementioned fluxes, the initial condition *X*_*3*_(*t*_*0*_) was also estimated. The bounds for the rate constants and kinetic orders were kept the same as above, while the initial concentration was bounded within [0, 5].

**Figure 6:**
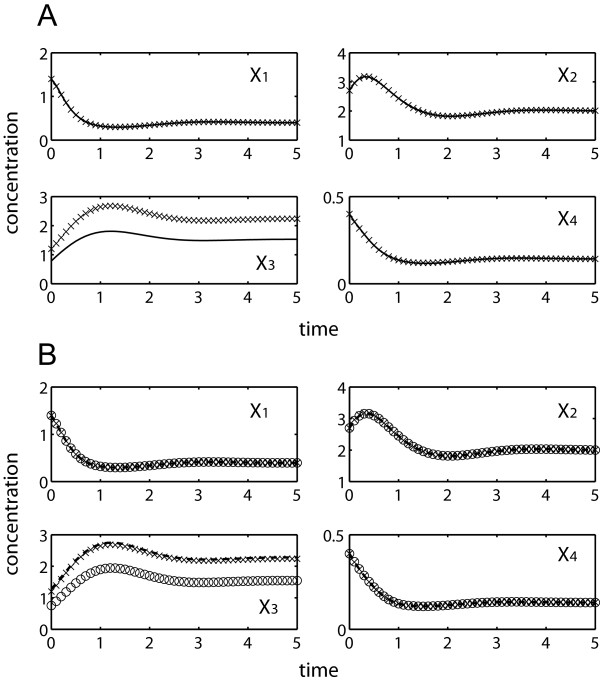
**Simultaneous and incremental estimation of the branched pathway with missing *X***_***3***_**: *in silico *noisy-free data (×).** (**A**) concentration predictions using parameter estimates from incremental method by Φ_C_ minimization (---); (**B**) concentration predictions using parameter estimates from simultaneous method (○) and proposed method (–––) by Φ_S_ minimization.

Table 3 summarizes the parameter estimation results. Four out of five repeated runs of Φ_C_ simultaneous optimization were again prematurely terminated after 5 days. Meanwhile, the rest of the estimations could provide reasonably good data fitting with the exception of fitting to *X*_*3*_ data as expected (see Figure 6). Like data noise, missing data led to increased inaccuracy of the parameter estimates, regardless of the estimation methods. Finally, the computational speedup by using the incremental over the simultaneous estimation was significant, but was lower than in the previous runs due to the additional integration of **X**_*U*_ and the larger number of independent parameters. The detailed values of the parameter estimates in this case study can be found in the Additional file 2: Tables S2 and S3.

**Table 3:** **Parameter estimations of the branched pathway model using noise-free data with *X***_***3 ***_**missing**

	**Simultaneous method**		**Incremental method**	
	$\text{min}\;\Phi_{C}^{a}$	**min Φ**_***S***_	**min Φ**_***C***_	**min Φ**_***S***_
CPU time (sec)	85.03 h	4002.01 ± 696.11	1404.22 ± 120.71	445.47 ± 35.94
eSSM GO iterations	308	365 ± 91	67 ± 10	48 ± 10
Parameter error (%)	71.90	43.50 ± 2.34	68.85 ± 4.57	40.47 ± 0.59
Φ_*C*_	4.54 × 10^-3^	6.46 × 10^-3^ ± 4.08 × 10^-4^	3.38 × 10^-3^ ± 1.14 × 10^-4^	5.94 × 10^-3^ ± 3.23 × 10^-5^
Φ_*S*_	1.03	2.99 × 10^-2^ ± 3.82 × 10^-4^	8.32 × 10^-2^ ± 4.04 × 10^-3^	2.94 × 10^-2^ ± 2.77 × 10^-6^

a. Only one out of five runs completed with a relative improvement of the objective function below 1% between iterations. The rest did not converge within the 5-day time limit after iterating for 471, 435, 863 and 786 times. The corresponding Φ_*C*_ at termination were 4.99× 10^-2^, 4.92 × 10^-2^, 1.17 × 10^-2^ and 1.57 × 10^-2^, respectively.

### The glycolytic pathway in *Lactococcus. lactis*

**Figure 7:**
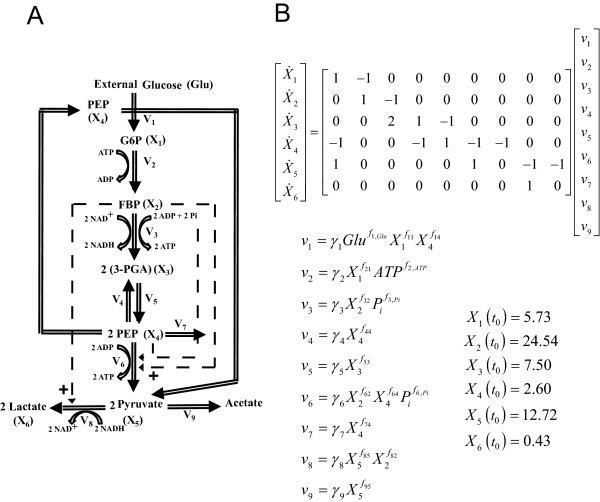
***L. lactis *glycolytic pathway.** (**A**) Metabolic pathway map (Double-lined arrows: flow of material; dashed arrows with plus or minus signs: activation or inhibition, respectively) and (**B**) the GMA model equations [16].

The second case study was taken from the GMA modeling of the glycolytic pathway in *L. lactis*[16], involving six internal metabolites: glucose 6-phosphate (G6P) – *X*_*1*_, fructose 1, 6-biphosphate (FBP) – *X*_*2*_, 3-phosphoglycerate (3-PGA) – *X*_*3*_, phosphoenolpyruvate (PEP) - *X*_*4*_, Pyruvate – *X*_*5*_, Lactate – *X*_*6*_, and nine metabolic fluxes. In addition, external glucose (Glu), ATP and Pi are treated as off-line variables, whose values were interpolated from measurement data. The pathway connectivity is given in Figure 7A, while the model equations are provided in Figure 7B.

**Figure 8:**
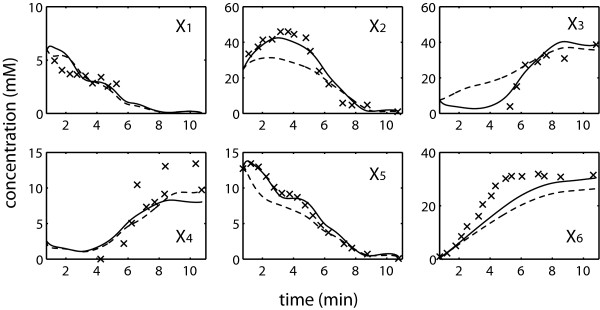
**Incremental estimation of the *L. lactis *model: Experimental data (×) compared with model predictions using parameters from concentration error minimization (–––) and slope error minimization (---).**

The time-course concentration dataset of all metabolites were measured using *in vivo* NMR [21,22], and smoothened data used for the parameter estimations below were shown in Figure 8. The raw data has been filtered previously [16], and these smoothened data for all metabolites but *X*_*6*_, were directly used for the concentration slope calculation in this case study. In the case of *X*_*6*_, a saturating Hill-type equation: *k*_*1*_*t*^*n*^ / (*k*_*2*_ *+ t*^*n*^) where *t* is time and the constants *k*_*1*_, *k*_*2*_, *n* are smoothing parameters, was fitted to the filtered data to remove unrealistic fluctuations. The central difference approximation was also adopted to obtain the time-slope data.

Fluxes *v*_*4*_, *v*_*7*_ and *v*_*9*_ were selected as the DOF, again to give the least number of **p**_*I*_ and to ensure that **S**_*D*_ is invertible. All rate constants were constrained to within [0, 50], while the independent and dependent kinetic orders were allowed within [0, 5] and [-5, 5], respectively. The difference between the bounds for the independent and dependent kinetic orders was done on purpose to simulate a scenario where the signs of the independent kinetic orders were known *a priori*.

Table 4 reports the outcome of the single-step and incremental parameter estimation runs using Φ_C_ and Φ_S_. The values of the parameter estimates are given in the Additional file 2: Table S4. Like in the previous case study, there was a significant reduction in the estimation runtime by using the proposed method over the simultaneous estimation, with comparable goodness of fit in concentration and slope. None of the five repeats of Φ_C_ simultaneous minimization converged within the five-day time limit, even after relaxing the convergence criteria of the objective function to 1%. On the other hand, the incremental estimation using Φ_C_ was not only able to converge, but was also faster than the simultaneous estimation of Φ_S_ that did not require any ODE integration. The incremental estimation using Φ_C_ was able to provide parameters with the best overall concentration fit (see Figure 8), despite having a large slope error. Finally, minimizing Φ_S_ does not guarantee that the resulting ODE is numerically solvable, as was the case of simultaneous estimation, due to numerical stiffness. But the incremental parameter estimation from minimizing Φ_S_ can produce solvable ODEs with good concentration and slope fits.

**Table 4:** **Parameter estimations of the *L. lactis *model**

	**Simultaneous method**		**Incremental method**	
	$\text{min}\;\Phi_{C}^{a}$	**min Φ**_***S***_	**min Φ**_***C***_	**min Φ**_***S***_
CPU time (sec)	>5 days	3476.89 ± 349.63	976.72 ± 31.01	20.82 ± 2.71
eSSM GO iterations	—	1662 ± 282	4 ± 1	33 ± 7
Φ_*C*_	—	Stiff ODE	2.20 ± 8.81 × 10^-3^	6.18 ± 7.28 × 10^-2^
Φ_*S*_	—	2.67 ± 1.93 × 10^-4^	1.51 × 10^3^ ± 52.50	5.79 ± 9.62 × 10^-4^

a. None of five runs finished with a relative improvement of the objective function below 1% within the 5-day time limit, after iterating for 60, 147, 93, 79 and 31 times. The corresponding Φ_*C*_ at termination were 9.31, 7.57, 8.77, 9.39 and 12.9, respectively.

## Discussion

In this study, an incremental strategy is used to develop a computationally efficient method for the parameter estimation of ODE models. Unlike most commonly used methods, where the parameter estimation is performed to minimize model residuals over the entire parameter space simultaneously, here the estimation is done in two incremental steps, involving the estimation of dynamic reaction rates or fluxes and flux-based parameter regressions. Importantly, the proposed strategy is designed to handle systems in which there exist extra degrees of freedom in the dynamic flux estimation, when the number of metabolic fluxes exceeds that of metabolites. The positive DOF means that there exist infinitely many solutions to the dynamic flux estimation, which is one of the factors underlying the parameter identifiability issues plaguing many estimation problems in systems biology [23,24].

The main premise of the new method is in recognizing that while many equivalent solutions exist for the dynamic flux estimation, the subsequent flux-based regression will give parameter values with different goodness-of-fit, as measured by Φ_C_ or Φ_S_. In other words, given any two dynamic flux vectors **v**(*t*_*k*_) satisfying ${\dot{X}}_{m}\left(t_{k}\right)=Sv\left(t_{k}\right)\text{,}$ the associated parameter pairs (**p**_*I*_, **p**_*D*_) may not predict the slope or concentration data equally well, due to differences in the quality of parameter regression for each **v**(*t*_*k*_). Also, because of the DOF, the minimization of model residuals needs to be done only over a subset of parameters that are associated with the flux degrees of freedom, resulting in much reduced parameter search space and correspondingly much faster convergence to the (global) optimal solution. The superior performance of the proposed method over simultaneous estimation was convincingly demonstrated in the two GMA modeling case studies in the previous section. The minimization of slope error, also known as slope-estimation-decoupling strategy method [7], is arguably one of the most computationally efficient simultaneous methods. In this strategy, the parameter fitting essentially constitutes a zero-finding problem and the estimation can be done without having to integrate the ODEs. Yet, the incremental estimation could offer more than two orders of magnitude reduction in the computational time over this strategy.

There are many factors, including data-related, model-related, computational and mathematical issues, which contribute to the difficulty in estimating kinetic parameters of ODE models from time-course concentration data [1]. Each of these factors has been addressed to a certain degree by using the incremental identification strategy presented in this work. For example, in data-related issues, the proposed method can be modified to handle the absence of concentration data of some metabolites, as shown in Figure 2. Nevertheless, the method is neither able nor expected to resolve the lack of complete parameter identifiability due to insufficient (dynamical) information contained in the data [23,24]. As illustrated in the first case study, single-step and incremental approaches provided parameter estimates with similar accuracies, which expectedly deteriorated with noise contamination and loss of data.

The appropriateness of using a particular mathematical formulation, like power law, is an example of model-related issues. As discussed above, this issue can be addressed after the dynamic fluxes are estimated, where the chosen functional dependence of the fluxes on a specific set of metabolite concentrations can be tested prior to the parameter regression [14]. Next, the computational issues associated with performing a global optimization over a large number of variables and the need to integrate ODEs have been mitigated in the proposed method by performing optimization only over the independent parameter subset and using a minimization of slope error, respectively. Finally, in this work, we have also addressed a mathematical issue related to the degrees of freedom that exist during the inference of dynamic fluxes from slopes of concentration data. However, extra degrees of freedom (mathematical redundancies) are also expected to influence the second step of the method, i.e. one-flux-at-a-time parameter estimation. For (log)linear regression of parameters in GMA models, such redundancy will lead to a lack of full column rank of the matrix containing the logarithms of concentration data **X**_*m*_(*t*_*k*_) and thus, can be straightforwardly detected.

The proposed estimation method has several weaknesses that are common among incremental estimation methods. As demonstrated in the first case study, the accuracy of the identified parameter relies on the ability to obtain good estimates of the concentration slopes. Direct slope estimation from the raw data, for example using central finite difference approximation, is usually not advisable due to high degree of noise in the typical biological data. Hence, pre-smoothing of the time-course data is often required, as done in this study. Many algorithms are available for such purpose, from simplistic polynomial regression and splines to more advanced artificial neural network [7,25] and Whittaker-Eilers smoother [26,27]. If reliable concentration slope estimates are not available, but bounds for the slope values can be obtained, then one can use interval arithmetic to derive upper and lower limits for the dependent fluxes and parameters using Equation (3) (or Equation (7) [28]. When the objective function involves integrating the model, validated solution to ODE with interval parameters can be used to produce the corresponding upper and lower bounds of concentration predictions [29]. Finally, the estimation can be reformulated, for example by minimizing the upper bound of the objective.

In addition to the drawback discussed above, the proposed strategy requires *a priori* knowledge about the topology of the network. For cellular metabolism, such information has become more readily available as genome-scale metabolic network of many important organisms, including human, *E. coli* and *S. cereviseae*, have been and are continuously being reconstructed [30]. For other networks, many algorithms also exist for the estimation of network topology based on time-series concentration data, including Bayesian network inference, transfer entropy, and Granger causality [31-33].

## Conclusions

The estimation of kinetic parameters of ODE models from time-course concentration data remains a key bottleneck in model building in systems biology. The lack of complete parameter identifiability has been blamed as the root cause of the difficulty in such estimation. In this study, a new incremental estimation method is proposed that is able to overcome the existence of extra degrees of freedom in the dynamic flux estimation from concentration slopes and to significantly reduce the computational requirements in finding parameter estimates. The method can also be applied, after minor modifications, to circumstances where concentration data for a few molecules are missing. While the present work concerns with the GMA modeling of metabolic networks, the estimation strategies discussed in this work have general applicability to any kinetic models that can be written as $\dot{X}\left(t_{k}\right)=Sv\left(t_{k}\right)\text{.}$ The creation of computationally efficient parameter estimation methods, such as the one presented here, represents an important step toward genome-scale kinetic modeling of cellular metabolism.

## Competing interest

The authors declare that they have no competing interests.

## Authors’ contributions

GJ conceived of the study, carried out the parameter estimation and wrote the manuscript. GS participated in the design of the study. RG conceived and guided the study and wrote the manuscript. All authors have read and approved the final manuscript.

## Funding

Singapore-MIT Alliance and ETH Zurich.

## Supplementary Material

Additional file 1 **Incremental Estimation Code.** Additional file 1 contains MATLAB codes for the parameter estimations in the two case studies: branched pathway model and *L. lactis* pathway model. Click here for file

Additional file 2 **Supplementary Tables.** Additional file 2 contains the parameter estimation results of the branched pathway model using noise-free data and analytical slopes, the parameter estimates of the two case studies, and the parameter estimation results of five repeated runs. Click here for file
