# Supplementary material for: Incremental parameter estimation of kinetic metabolic network models
Source: BMC Syst Biol. 2012 Nov 21;6:142. doi: 10.1186/1752-0509-6-142 (PMC3568022; doi:10.1186/1752-0509-6-142)
Supplement: Additional file 2 — Supplementary Tables. Additional file 2 contains the parameter estimation results of the branched pathway model using noise-free data and analytical slopes, the parameter estimates of the two case studies, and the parameter estimation results of five repeated runs. [file 1752-0509-6-142-S2.pdf]

Incremental parameter estimation of kinetic metabolic network models

Supplementary material

Gengjie Jia, Gregory N. Stephanopoulos and Rudiyanto Gunawan

Table S1 - Parameter estimations of the branched pathway model using noise-free data and analytical slope values

	Simultaneous method		Incremental method	
	$\min \Phi_C$	$\min \Phi_S$	$\min \Phi_C$	$\min \Phi_S$
CPU time (sec) [a]	56.00 hours [b]	785.79 [c] ±50.80 [c]	256.43 ±16.42	3.17 $\pm 4.72\times10^{-2}$
eSSM GO iterations	323	5051 ± 307	19 ± 4	29 ± 4
Parameter error	49.10%	4.93×10^{-3}% $\pm 2.84\times10^{-4}$%	3.01×10^{-5}% $\pm 4.61\times10^{-6}$%	1.96×10^{-5}% $\pm 7.63\times10^{-7}$%
Φ_C [d]	<u>4.54×10^{-3}</u>	2.17×10^{-6} $\pm 4.00\times10^{-9}$	<u>4.48×10^{-9}</u> $\pm 3.31\times10^{-11}$	6.75×10^{-9} $\pm 1.19\times10^{-10}$
Φ_S [d]	4.84×10^{-2}	<u>3.01×10^{-6}</u> $\pm 2.57\times10^{-9}$	2.66×10^{-8} $\pm 9.68\times10^{-10}$	<u>1.36×10^{-8}</u> $\pm 6.17\times10^{-11}$

a. CPU time was based on a workstation with dual Intel Quad-Core 2.83 GHz processors.

b. Only one out of five runs was stopped with relative improvement of the objective function below 1% between iterations. The rest did not converge within the 5-day time limit after iterating for 583, 989, 777, and 661 times. The corresponding Φ_C at termination were 4.85×10^{-2}, 1.39×10^{-2}, 1.75×10^{-2} and 3.75×10^{-2}, respectively.

c. Mean value and standard deviation (±) out of five runs.

d. Root mean square error of model predictions, where the underlined value refers to the objective function of the minimization.

Table S2 - Parameter estimates of the branched metabolic pathway model (simultaneous method)

*	True Values (Voit and Almeida, 2004)	Simultaneous method $\min \Phi_S$			
		(1)	(2)	(3)	(4)
γ_1	20	19.9999	22.0151	23.2163	20.9692
f_{13}	0.8	0.8000	0.6179	0.2340	0.3713
γ_2	8	7.9998	10.1122	6.5743	9.8968
f_{21}	0.5	0.5000	0.3498	0.5569	0.3599
γ_3	3	2.9998	5.1168	2.3392	4.9036
f_{32}	0.75	0.7500	0.5174	0.7568	0.5342
γ_4	5	4.9998	7.0401	2.7497	9.3560
f_{43}	0.5	0.5000	0.3262	0.4526	0.3054
f_{44}	0.2	0.2000	0.1135	0.0031	0.2082
γ_5	2	2.0002	1.5302	7.6821	4.2064
f_{51}	0.5	0.4999	0.8258	0.0003	0.1642
γ_6	6	5.9997	7.7990	8.4180	6.4270
f_{64}	0.8	0.7999	1.2250	0.0452	0.2945
$X_3(t_0)$	1.2	—	—	—	0.7548

* This table reports the parameter estimates with the minimal objective function value out of five runs.

(1) using noise-free data and analytical slopes; (2) using noise-free data; (3) using noisy data; (4) using noise-free data with missing X_3

Table S3 - Parameter estimates of the branched metabolic pathway model (incremental method)

*	True Values (Voit and Almeida, 2004)	Incremental method $\min \Phi_C$				Incremental method $\min \Phi_S$			
		(1)	(2)	(3)	(4)	(1)	(2)	(3)	(4)
γ_1	20	20.0000	20.0105	24.9989	13.4674	20.0000	22.5904	15.0593	24.9585
f_{13}	0.8	0.8000	0.7634	0.3366	1.0920	0.8000	0.6058	0.7824	0.4894
γ_2	8	8.0000	8.7730	14.1896	7.4143	8.0000	10.3220	7.2424	10.1723
f_{21}	0.5	0.5000	0.4410	0.2610	0.5301	0.5000	0.3417	0.4804	0.3479
γ_3	3	3.0000	3.6749	8.6709	2.5980	3.0000	5.2978	2.8968	5.1604
f_{32}	0.75	0.7500	0.6680	0.3577	0.8098	0.7500	0.5072	0.6827	0.5160
γ_4	5	5.0000	5.9268	10.9451	8.2781	5.0000	7.2630	3.4761	7.0669
f_{43}	0.5	0.5000	0.4021	0.1585	0.8642	0.5000	0.3213	0.4371	0.3023
f_{44}	0.2	0.2000	0.1719	0.0579	0.4950	0.2000	0.1133	0.0338	0.1042
γ_5	2	2.0000	1.3828	0.3694	1.6768	2.0000	1.6284	0.8468	3.2351
f_{51}	0.5	0.5000	0.8068	0.0000	1.2353	0.5000	0.7753	1.4665	0.2243
γ_6	6	5.9999	7.3216	1.4041	15.0425	6.0000	7.7068	11.1042	5.7002
f_{64}	0.8	0.8000	1.2352	0.6459	1.7137	0.8000	1.1649	2.0000	0.3960
$X_3(t_0)$	1.2	—	—	—	0.7865	—	—	—	1.2773

* This table reports the parameter estimates with the minimal objective function value out of five runs.

(1) using noise-free data and analytical slopes; (2) using noise-free data; (3) using noisy data; (4) using noise-free data with missing X_3

Table S4 - Parameter estimates of the *L. lactis* metabolic model

*	Simultaneous method	Incremental method	
	$\min \Phi_S$	$\min \Phi_C$	$\min \Phi_S$
γ_1	2.2638	9.7891	0.4994
$f_{1, Glu}$	0.0690	0.2627	0.8716
f_{11}	1.2991	0.0309	-1.0343
f_{14}	-0.5461	0.3979	0.9642
γ_2	0.2330	49.9072	49.9999
f_{21}	1.9573	0.4358	0.4404
$f_{2, ATP}$	0.9219	-0.3360	-0.8733
γ_3	5.8716	8.3470	5.9069
f_{32}	0.2739	0.4571	0.3602
$f_{3, Pi}$	-0.1315	0.1254	0.0477
γ_4	1.5800×10^{-13}	49.6053	0.4193
f_{44}	8.9194×10^{-6}	4.9730	1.7635
γ_5	49.9999	5.2494	49.9999
f_{53}	-0.4609	3.4524	-0.0887
γ_6	3.3189	11.0241	8.2447
f_{62}	0.4006	0.3926	0.2874
f_{64}	0.1383	0.0208	0.2041
$f_{6, Pi}$	-0.2920	0.0279	-0.2545
γ_7	0.0001	0	3.0295×10^{-5}
f_{74}	4.9999	1.0855×10^{-7}	0.0005
γ_8	6.3648×10^{-9}	0.5332	0.5332
f_{85}	1.7507	0.1781	0.1781
f_{82}	4.4842	0.4804	0.4804
γ_9	5.4359	34.4010	17.7804
f_{95}	0.5957	0.4394	0.3410

* This table reports the parameter estimates with the minimal objective function value out of five runs.

REFERENCES

1. Voit, E.O. and Almeida, J. (2004) Decoupling dynamical systems for pathway identification from metabolic profiles, *Bioinformatics*, **20**, 1670-1681.
